# NanoSIMS Imaging Reveals the Impact of Ligand-ASO Conjugate Stability on ASO Subcellular Distribution

**DOI:** 10.3390/pharmaceutics14020463

**Published:** 2022-02-21

**Authors:** Emma Kay, Rouven Stulz, Cécile Becquart, Jelena Lovric, Carolina Tängemo, Aurélien Thomen, Dženita Baždarević, Neda Najafinobar, Anders Dahlén, Anna Pielach, Julia Fernandez-Rodriguez, Roger Strömberg, Carina Ämmälä, Shalini Andersson, Michael Kurczy

**Affiliations:** 1 Mechanistic and Structural Biology, Discovery Sciences, BioPharmaceuticals R&D, AstraZeneca, SE-431 83 Gothenburg, Sweden; emma.kay@astrazeneca.com; 2 Department of Biosciences and Nutrition, Karolinska Institutet, SE-141 83 Huddinge, Sweden; rouven.stulz@astrazeneca.com (R.S.); roger.stromberg@ki.se (R.S.); 3 Oligonucleotide Discovery, Discovery Sciences, BioPharmaceuticals R&D, AstraZeneca, SE-431 83 Gothenburg, Sweden; anders.dahlen@astrazeneca.com (A.D.); shalini.andersson@astrazeneca.com (S.A.); 4 DMPK, Early Cardiovascular, Renal and Metabolism, BioPharmaceuticals R&D, AstraZeneca, SE-431 83 Gothenburg, Sweden; cecile.becquart@astrazeneca.com (C.B.); jelena.lovric@astrazeneca.com (J.L.); 5 Department of Chemistry and Molecular Biology, University of Gothenburg, SE 412 96 Gothenburg, Sweden; aurelien.thomen@chem.gu.se; 6 Discovery Biology, Discovery Sciences, BioPharmaceuticals R&D, AstraZeneca, SE-431 83 Gothenburg, Sweden; carolina.tangemo@astrazeneca.com; 7 Bioscience, Early Respiratory and Immunology, BioPharmaceuticals R&D, AstraZeneca, SE-431 83 Gothenburg, Sweden; dzenita.bazdarevic1@astrazeneca.com; 8 Medicinal Chemistry, Research and Early Development, Respiratory and Immunology (R&I), BioPharmaceuticals R&D, AstraZeneca, SE-431 83 Gothenburg, Sweden; neda.najafinobar@astrazeneca.com; 9 Centre for Cellular Imaging, Sahlgrenska Academy, University of Gothenburg, SE-405 30 Gothenburg, Sweden; anna.pielach@bioenv.gu.se (A.P.); julia.fernandez-rodriguez@cci.sahlgrenska.gu.se (J.F.-R.); 10 Bioscience, Early Cardiovascular, Renal and Metabolism, BioPharmaceuticals R&D, AstraZeneca, SE-431 83 Gothenburg, Sweden; carina.ammala@astrazeneca.com

**Keywords:** NanoSIMS, mass spectroscopy imaging, targeted delivery, antisense oligonucleotide

## Abstract

The delivery of antisense oligonucleotides (ASOs) to specific cell types via targeted endocytosis is challenging due to the low cell surface expression of target receptors and inefficient escape of ASOs from the endosomal pathway. Conjugating ASOs to glucagon-like peptide 1 (GLP1) leads to efficient target knockdown, specifically in pancreatic β-cells. It is presumed that ASOs dissociate from GLP1 intracellularly to enable an ASO interaction with its target RNA. It is unknown where or when this happens following GLP1-ASO binding to GLP1R and endocytosis. Here, we use correlative nanoscale secondary ion mass spectroscopy (NanoSIMS) and transmission electron microscopy to explore GLP1-ASO subcellular trafficking in GLP1R overexpressing HEK293 cells. We isotopically label both eGLP1 and ASO, which do not affect the eGLP1-ASO conjugate function. We found that the eGLP1 peptide and ASO are not detected at the same level in the same endosomes, within 30 min of GLP1R-HEK293 cell exposure to eGLP1-ASO. When we utilized different linker chemistry to stabilize the GLP1-ASO conjugate, we observed more ASO located with GLP1 compared to cell incubation with the less stable conjugate. Overall, our work suggests that the ASO separates from GLP1 relatively early in the endocytic pathway, and that linker chemistry might impact the GLP1-ASO function.

## 1. Introduction

Antisense oligonucleotides (ASOs) are being explored as nucleic acid therapeutics and are taken up by permissive cell lines via endocytosis. This can downregulate the expression of specific RNAs and, thus, ASOs are of interest for modulating drug targets that cannot be modulated with small molecules or antibodies [1,2,3]. Gapmer ASOs enable RNA knockdown by sequence-dependent cleavage, mediated by RNase H1 endonuclease [4]. Therapeutic gapmer ASOs are typically extensively modified to improve the binding affinity, while reducing cellular toxicity [4,5,6]. The inclusion of phosphorothioate (PS) linkages between nucleotides improves the nuclease resistance and increases binding to serum proteins [7]; however, the productive cellular uptake of PS-ASOs is still poor in most cell types.

Conjugation to targeting ligands is employed to improve the PS-ASO uptake in nonpermissive cell types and/or to promote uptake by disease-relevant cells. For example, N-acetylgalactosamine (GalNAc) has been employed and studied extensively as a ligand for targeting PS-ASOs to hepatocytes via binding to asialoglycoprotein receptors (ASGPRs) [8]. We also previously described the use of ligands targeting the glucagon-like peptide 1 receptor (GLP1R) for the specific targeting of PS-ASOs to pancreatic beta cells, which poorly internalize PS-ASOs. GLP1R is a G-protein-coupled receptor (GPCR), which is predominantly expressed on pancreatic β cells [9,10] and undergoes rapid agonist-mediated internalization into endosomes [11,12,13,14,15]. We conjugated ASOs to an engineered GLP1 (eGLP1), which had been previously utilized to deliver estrogen [16], and we observed an increased target RNA knockdown in GLP1R-expressing cells compared to treatment with unconjugated ASO c10 [17].

Targeting PS-ASOs to the right cells is not the only challenge to be overcome in their development as therapeutics. Once inside the cell, PS-ASOs typically accumulate in endosomes, from which they need to escape in order to interact with their target RNA, which occurs very inefficiently [18]. We previously observed fluorescently labelled eGLP1-ASO to accumulate in punctate cytoplasmic structures, presumably endosomes, in HEK293 cells over-expressing GLP1R (GLP1R-HEK293 cells). Since the knockdown of the target RNA was also observed in these cells, we presume that some of the delivered PS-ASO escaped the endosome, but this is difficult to observe using fluorescence microscopy [19]. To better understand the relationship between PS-ASO subcellular trafficking and target RNA knockdown following targeted delivery, it would be informative to be able to quantify the distribution of both the PS-ASO and the targeting ligand at the subcellular level. 

The relative quantitation of ASOs in the nucleus and cytoplasm of HeLa cells has been achieved with fractionation and ELISA [20]. One route to performing the subcellular quantification of isotopically or atomically labeled molecules is to use nanoscale secondary ion mass spectrometry (NanoSIMS) correlated to electron microscopy (EM). We previously described both subcellular mapping and the quantitation of ^13^C-labelled dopamine in secretory vesicles using NanoSIMS and EM [21]. NanoSIMS with correlative EM has also recently been employed for the relative quantitation of labeled PS-ASOs in subcellular compartments [22], but the detection and quantitation of both a targeting ligand and PS-ASO at the subcellular level has not yet been described. Here, we use NanoSIMS to detect PS-ASO conjugates incorporating different isotopic labels on the conjugating peptide and the PS-ASO. This enables the mapping and quantification of both a PS-ASO and its targeting ligand in subcellular organelles for the first time.

## 2. Materials and Methods

### 2.1. Mammalian Cell Culture

HEK293 cells stably transfected with GLP1R (GLP1R-HEK293 cells) were maintained in Dulbecco’s Modified Eagle’s Medium (Thermo Fisher Scientific, Waltham, MA, USA ) supplemented with 10% heat-inactivated fetal bovine serum (FBS) (Thermo Fisher Scientific, Waltham, MA, USA) and 1% hygromycin B, at 37 °C under 5% CO_2_.

### 2.2. Hepatic Spheroid Formation and Maintenance

Cryopreserved primary human hepatocytes were obtained from BioIVT (Brussels, Belgium) and thawed according to the supplier’s instructions. Briefly, the hepatocytes were thawed at 37 °C and added to prewarmed seeding medium (Williams’ E medium without glucose supplemented with 1 g/L glucose, 2 mM GlutaMAX, 100 units/mL penicillin, 100 μg/mL streptomycin, 0.1 nM insulin, 5.5 μg/mL transferrin, 6.7 ng/mL sodium selenite, 100 nM dexamethasone, and 10% FBS). In total, 2000 viable cells per well were seeded into ultra-low adhesion 96-well plates (Corning, Wiesbaden, Germany) (only the 600 inner wells were used). Plates were then centrifuged at 200× *g* for 2 min to induce the spheroids formation by self-aggregation and incubated at 37 °C, under 5% CO_2_. The medium was changed to FBS-free medium every 24 h from day 4 to 7 post seeding, thereafter every 48 h.

### 2.3. Synthesis of Peptides, PS-ASOs, and eGLP1-ASO Conjugates

All starting materials, reagents, and solvents were used as received. Unless otherwise stated, solvents and reagents were obtained from Sigma-Aldrich(St.Louis, MO, USA. Phase separators were obtained from Biotage(Uppsala, Sweden). The ^1^H and ^13^C-NMR spectra were recorded at 300 K on a Bruker 500 MHz system equipped with a CryoProbe, operating at 500 and 126 MHz, respectively. Chemical shifts were recorded in ppm relative to the solvent residual signals: CDCl_3_.

#### 2.3.1. Malat1 PS-ASO Synthesis

PS-ASOs targeting *Malat1* were synthesized on a 32 μmol scale on an ÄKTA Oligopilot 10 system by using commercially available DNA phosphoramidate building blocks and PS 5G UnyLinker support (GE Healthcare). Detritylation was performed by using 3% dichloroacetic acid in toluene. 5-(Benzylthio)-1H-tetrazole (BTT) was used as activating agent. CE backbone deprotection was performed with diethylamine/toluene (20% *v*/*v*). Phosphoramidites were dissolved to a final concentration to 0.1 M in anhydrous acetonitrile prior to use. The ^34^S- or ^32^S-PADS was dissolved in 1:1 (*v*/*v*) MeCN/3-picoline (0.2 M) and aged for at least 16 h before use. Recirculation times for phosphoramidites were 5 min for DNA building blocks, 10 min for cEt building blocks, and 40 min for the MMT-hexylamine building block. No detritylation was performed after attachment of the 5′ Hexylamine. ASOs were cleaved from the solid support by treatment with aqueous ammonia (26%) at 55 °C for 15–20 h. LC–MS was performed either on an XBridge C_18_ column by using mixtures of 0.1 m triethylammonium acetate (pH 7)/acetonitrile or on the same column by using mixtures of 0.1 M ammonium carbonate (pH 7.6)/methanol. ASO and conjugates were purified on an XBridge C_18_, 5 μm 19 × 150 mm column by using gradients from 0.1 M ammonium carbonate (pH 7.6) to methanol. The pure fractions were evaporated, dissolved in 5 mL water, and the pH was adjusted to 4.5 with acetic acid. Then, the solution was warmed to 40 °C for 1h, the pH was adjusted to 6 with 3 M NaOH, and then a solution of 3 M NaAc (pH 5) was added, followed by 15 mL of EtOH. The solution was cooled to −20 °C for 1 h and then centrifuged. The supernatant was discarded, and the precipitate was washed with ice-cold EtOH, diluted with water, and freeze dried.

#### 2.3.2. GalNAc Conjugate Synthesis

The ^34^S-labeled hexylamine oligo (224.5 mg, 0.03 mmol) was dissolved in 2.25 mL 0.06 M borate buffer pH 9.6. GalNAc PFP (255 mg, 0.13 mmol) was dissolved in 3.6 mL DMSO, added, and the reaction mixture was stirred at room temperature. After 60 min reaction time, the pH of the reaction mixture was adjusted to 10. After another 60 min, the pH was adjusted to 9.5 with acetic acid and the reaction mixture was diluted with 10 mL of 26% aq. NH_3_ was stirred overnight and freeze dried. The resulting gel was diluted with water and purified on a 20 g ISOLUTE weak anion exchange (NH_2_) cartridge by eluting with a gradient from 0 to 50% MeCN in 1 M Triethylammonium carbonate buffer (pH 8). The fractions containing oligonucleotide were freeze dried and purified by reverse-phase HPLC and freeze dried to provide a white powder.

#### 2.3.3. eGLP1 Peptide Synthesis

Automated peptide synthesis was carried out on a Biotage Alstra peptide synthesizer using Fmoc/t-Bu chemistry. Peptides were synthesized on ChemMatrix Tetagel resin using DIC/oxyma as coupling agents and 20% piperidine in DMF for FMOC deprotection. Couplings were conducted at 75 °C for 5 min for all amino acids except cysteine and histidine, which were coupled for 20 min at 40 °C. Single couplings were employed for the first 20 amino acids and double couplings for following residues. Fmoc-2,5,Diiodotyrosine was used without phenol protection and double coupled. After the synthesis was finished, the resin was washed with DMF, MeOH, and DCM (×3 each), followed by a wash with diethyl ether. Then, the resin was dried under vacuum. The peptide was cleaved by shaking the resin with 10 mL of a mixture of TFA/TIS/DODT/H2O (95,5,2.5,5) for 3 h. The resin was filtered off and the filtrate directly drained into 80 mL of cold diethyl ether. The precipitate was collected by centrifugation, resuspended in cold diethyl ether and again collected by filtration. The resulting white solid was freeze dried and purified by reverse-phase HPLC using gradients from water containing 0.15% TFA to acetonitrile.

#### 2.3.4. ASO eGLP1 Peptide Conjugations Using Maleimide

5′-Hexylamine-modified ASO was dissolved in 0.1 M potassium phosphate buffer (350 μL; pH 7.1). 3-(Maleimido)propionic acid N-hydroxysuccinimide (8 equivalents) was dissolved in DMSO (300 μL) and added to the ASO solution. The reaction mixture was left to stand at room temperature for 1 h. The oligo was precipitated from sodium acetate/ethanol (1:4; 0.3 M). The precipitate was redissolved in a minimal amount of 0.1 M potassium phosphate buffer (0.2 mL; pH 7.1) and then the GLP1-cysteine peptide, dissolved in a minimal amount of water:acetonitrile 1:1, was added. The reaction mixture was left to stand at room temperature for 4 h, then freeze dried and purified by reverse-phase HPLC.

#### 2.3.5. ASO eGLP1 Peptide Conjugation Using Strain-Promoted Azide Alkyne Click Reaction

The hexylamine-modified oligonucleotide was dissolved in 200 µL phosphate buffer pH 7.4 (0.1 M) and 200 µL MeCN. ((1R,8S,9s)-bicyclo[6.1.0]non-4-yn-9-yl)methyl (4-nitrophenyl) carbonate (7eq. by mass) in 200 µL MeCN and 200 µL phosphate buffer were added. In total, 0.1 M NaOH was added until the reaction mixture was deeply yellow. After standing at room temperature overnight, the reaction mixture was freeze dried and purified by passing the reaction mixture through a 10 g ISOLUTE NH_2_ Anion exchange column using a gradient from MeCN to 60% TEAC buffer (Triethylammonium carbonate, 1 M, pH 7.2). The fractions containing product were freeze dried to provide a white powder. The bicyclononyne-modified ASO was then dissolved in triethylammonium acetate buffer (pH 7) and the azide-bearing peptide (1.5 equivalents) was added as a solution in 1:1 MeCN:water. The reaction mixture was left standing overnight, freeze dried, and purified by reverse-phase HPLC. Pure fractions were freeze dried to provide pure conjugate as a white powder.

### 2.4. GLP1R Internalization DiscoverX Assay

GLP1R internalization in U2OS cells was measured with the PathHunter^®^ eXpress GLP1R Activated GPCR Internalization Assay kit (DiscoverX, #93-0724E3CP0L) using the manufacturer’s protocol as previously described [23]. Cells were incubated with compounds for 30 min at 37 °C, under 5% CO_2_.

### 2.5. ASO Incubations

For immunofluorescence microscopy, GLP1R-HEK293 cells were seeded at a density of 25,000 cells/well in 96-well imaging plates (Greiner Bio-One) coated with poly-D-lysine. For TEM and NanoSIMS, GLP1R-HEK293 cells were seeded at a density of 200,000-400,000 cells/mL in 35 mm glass-bottomed dishes (MatTek) coated with 5 μg/mL poly-D-lysine (Sigma-Aldrich, St. Louis, MO, USA). Twenty-four hours later, the cells were washed once with Optimem + Glutamax (Thermo Fisher Scientific, Watham, MA, USA) + 0.1% bovine serum albumin (BSA) (Sigma-Aldrich, St. Louis, MO, USA), and then incubated with Optimem + 0.1% BSA for 15 min at 37 °C. Compounds were diluted in Optimem + 0.1% BSA and cell incubations were performed at 37 °C for the timepoints indicated. The compounds were removed and the cells were washed three times with phosphate-buffered saline (PBS) prior to fixation for immunofluorescence microscopy or electron microscopy/NanoSIMS as described below. Spheroids were treated with GalNAc-^34^S-labeled-*Malat1* diluted in maintenance medium at day 12 postseeding. Twenty-four hours later, spheroids were collected and fixed for electron microscopy and NanoSIMS analysis as described below.

### 2.6. Immunofluorescence Microscopy

Following compound incubation, cells in 96-well plates were fixed in 3.7% formaldehyde (Sigma-Aldrich, St. Louis, MO, USA) in PBS for 10 min at room temperature. The cells were then washed three times with PBS and blocked with PBS + 2% BSA (Sigma-Aldrich) + 0.05% saponin (FLUKA) for 2 h at 4 °C. Primary antibody incubations were performed with anti-GLP1R (Mab7F38 from Developmental Studies Hybridoma Bank, described in [24]), rabbit anti-early endosome-associated antigen 1 (EEA1) (Cell Signaling, Danvers, MA, USA) #C45B10) or rabbit anti-LAMP1 (Cell Signaling, Danvers, MA, USA) antibodies diluted in PBS + 2% BSA + 0.05% saponin overnight at 4 °C. Cells were then washed three times with PBS + 0.05% saponin and incubated with Alexa Fluor conjugated secondary antibodies (Life Technologies, Carlsbad, CA, USA), diluted in PBS + 2% BSA + 0.05% saponin, for 2 h at room temperature. The cells were then washed three times with PBS + 0.05% saponin and incubated with DAPI (Sigma-Aldrich, St. Louis, MO, USA) to counterstain the nuclei and, in some experiments, CellMask Deep Red (Life Technologies, Carlsbad, CA, USA) was used as a counterstain for the whole cell. Images were acquired on a Yokogawa CV7000 spinning disk confocal microscope with a 60× water objective, with at least 8 fields of view acquired from duplicate wells for each condition acquired.

### 2.7. Quantitative RT-PCR

GLP1R-HEK293 cells were seeded at a density of 200,000 cells/mL per well in DMEM + 10% FBS (Life Technologies, Carlsbad, CA, USA) in poly-D-lysine-coated 384-well plates (Corning) and incubated for 24 h at 37 °C, under 5% CO_2_. eGLP1-ASO conjugates diluted in culture media were incubated with GLP1R-HEK293 cells for 24 h at 37 °C, under 5% CO_2_. The cells were then washed once with cold DPBS (Life Technologies) and then lysed with RLN lysis buffer (QIAGEN) with 4% RNAsecure Inactivation Reagent (Thermo Fisher Scientific, Waltham, MA, USA). Lysates were then transferred to the corresponding wells of a 384-well PCR plate (Axygen, Union City, CA, USA) containing cDNA synthesis master mix (Thermo Fisher Scientific). The reverse transcription reaction was performed at 37 degrees for 60 min, and then stopped by heating at 95 degrees for 5 min. For real time quantitative PCR, the resulting cDNA samples were diluted and transferred, using an Echo 655 liquid handler, to a PCR master mix solution consisting of Taqman Gene Expression Master mix (Applied Biosystems, Waltham, MA, USA) and Malat1 (Hs00273907_s1) or Hprt1 (Hs02800695_m1) PCR primer sets (Thermo Fisher Scientific) diluted in RNAse-free water. The amplification reaction and analysis were performed on QuantStudio 7 Flex real-time PCR machine with the following conditions: 50 °C for 2 min, 95 °C for 10 min, followed by 40 cycles of 95 °C for 15 s, and 60 °C for 1 min. Relative mRNA expression levels for *Malat1* ($2^{-\Delta C_{q}}$) were calculated by normalizing the *Malat1* expression levels to those of the reference gene *HPRT1*. The $2^{-\Delta C_{q}}\;$values for *Malat1* derived from cells treated with ASOs were normalized to untreated controls.

### 2.8. Electron Microscopy

Following compound incubation, cells in 35 mm dishes were washed three times with PBS and then fixed with a modified Karnovsky’s fixative (2.5% glutaraldehyde (Agar Scientific, Essex, UK), 2% formaldehyde (Sigma-Aldrich, St. Louis, MO, USA), 0.02% sodium azide (BDH, Poole, UK) in 0.05M Na-cacodylate buffer) for 30 min at room temperature. The dishes were then washed twice with Ca^2+^ and Mg^2+^-free PBS (Thermo Fisher Scientific), once with 50 mM glycine (Sigma-Aldrich) in PBS, and then twice with PBS. The dishes were then incubated for 1 h with 1% OsO_4_ (Sigma-Aldrich) in 0.05 M Na-cacodylate buffer and then washed at least 3 times with H_2_O. The dishes were then incubated for 20 min with 1% tannic acid and washed three times with H_2_O. The dishes were then incubated for 15 min with 1% uranyl acetate (Merck, Darmstadt, Germany Sigma-Aldrich, St. Louis, MO, USA) in the dark and washed three times with H_2_O. The cells were then sequentially dehydrated with 50%, 70%, 85%, 95%, and absolute ethanol (5 min incubation with each). Embedding was performed in Agar 100 premix kit hard (Agar Scientific, Essex, UK). Agar 100 epoxy resin was mixed with premixed hardeners (DDSA and MNA), but not with accelerator (BDMA), for 30 min to produce mixture A. Mixture A was mixed with accelerator (BDMA) for 30 min to produce mixture B. The final composition of this medium hardness resin was agar resin 46.6%, hardeners 50.5%, and BDMA 2.9%. Each dish was coated with 50% mixture A and 50% absolute ethanol and incubated for 30 min. Dishes were then incubated for 5 min and then 10 min with mixture A. Each dish was then incubated for 15 min with mixture B. BEEM capsules were filled with 100% mixture B. Resin was decanted from MatTek dishes (MatTek, Ashland, MA, USA) and the dishes were inverted onto BEEM capsules(Agar Scientific, Essex, UK), so that the cell monolayer was covering the mouth of the capsule. MatTek dishes with BEEM capsules were then carefully inverted so that the BEEM capsule sat top of the dish. The tube and dish were then inverted together and polymerized at 60 °C for 45 h. After polymerization, resin blocks were dislodged from MatTek dishes with liquid nitrogen and sectioned with an ultramicrotome (Leica EM UC6) at desired thickness.

Spheroid samples were prepared as described above with some modifications to the protocol. Briefly, the collected spheroids were placed in the modified Karnovsky fixative for 1 h at 4 °C, then washed twice with 100 mM sodium cacodylate buffer, once with 50 mM glycine in 100 mM sodium cacodylate buffer, and twice with 100 mM sodium cacodylate buffer. Samples were post fixed with 1% osmium tetroxide at 4 °C for 1 h in the dark, followed by 1% tannic acid for 20 min at room temperature in the dark, and 1% uranyl acetate for 40 min at room temperature in the dark. Spheroids were dehydrated with increasing concentration of ethanol (30, 50, 70, 85, 95, and 100%) and embedded in Agar 100 resin.

Sections were prepared at 300 nm thickness on EM finder grids (TEM-FCF200CU50, Sigma-Aldrich, St Louis, MO, USA). TEM micrographs were acquired using a Talos L120C transmission electron microscope (FEI) at 2000–3400× magnification. Lower magnification views of the finder grid position were acquired to enable imaging of the same cells by both TEM and NanoSIMS.

### 2.9. NanoSIMS Imaging

Analysis was performed at the Chemical Imaging Infrastructure at Chalmers University of Technology and University of Gothenburg with a NanoSIMS 50L (CAMECA, Gennevilliers, France). Spheroid samples were gold-coated and a 16 keV Cs^+^ primary ion beam of 8 pA (D1.2) was used to raster the sample surface. The detectors were tuned to measure ^13^C^12^C-, ^13^C^14^N-, ^32^S-, and ^34^S- secondary ions. Prior to imaging, the areas of interest were implanted to obtain a fluence of 10^17^ Cs^+^.cm^−2^ to reach sputtering steady state (as a result, around 200 nm of sample was sputtered away). Images were acquired on field size of 25 × 25, 20 × 20, 15 × 15 μm^2^ with an image resolution of 256 × 256 pixels and a dwell time of 5ms.pixel^−1^. At least 10 planes were accumulated for each sample where the dimensions of an individual plane were between 100 and 60 nm^2^ depending on the field of view (FOV). GLP1R-HEK293 cells received the implantation dose 10^17^ Cs^+^.cm^−2^ and were analyzed with either 2 pA primary ion current with a 40 ms dwell time or an 8 pA ion current and a 5 ms dwell time. The detectors were tuned to measure ^13^C^12^C-, ^13^C^14^N-, ^32^S-, ^34^S-, I ^127^I secondary ions.

### 2.10. Image Analysis

NanoSIMS images were visualized and analyzed with the WinImage software (CAMECA, Gennevilliers, France) or the OpenMIMS plugin of FIJI/ImageJ (version 3 0.5—rev 1, National Institutes of Health). Sequential image planes were collected, drift corrected, and accumulated to increase ion counts and reduce statistical error. The ^34^S-/^32^S- ratio maps were extracted. Seven treated hepatocytes were imaged and regions of interest (ROIs) were determined based on the ^34^S-/^32^S- ratio. ROIs were defined manually. The different subcellular compartments were defined based on the CN image, the ^34^S hotspots on the ^34^S/^32^S image, and the ^32^S hotspots based on the ^32^S image. ROIs overlapping in the ^34^S/^32^S and ^32^S images were excluded.

The ^34^S^-^/^13^C^12^C^−^ (ASO) images were generated in Image J by multiplying the average sulfur isotopic ratio by the ^32^S image and subtracting it from the ^34^S image using the image calculation function. The image was divided by the number of ^34^S labels on the ASO and then normalized to the ^13^C^12^C image. The ^127^I/^13^C^12^C (eGLP1) images were generated by dividing the ^127^I image by the number of ^127^I labels and normalizing to ^13^C^12^C. ROI data were generated in the same way, except that the images were first binned to pixel bins equal to 468 × 468 nm. ROIs were selected for analysis based on the ^127^/^13^C^12^C signal, provided that the value of the ROI was higher than 3 times the standard deviation of all the ROIs in the image(3 σ).

The ImageJ plugin BigWarp was used to register NanoSIMS images with their matching TEM images [25]. Landmarks for the image registration were marked on ^12^C/^14^N images (not shown) and then the same landmarks were applied to the ^127^I_2_-eGLP1 and ^34^S-ASO images from the same acquisition, resulting in unbiased registration of these images with the TEM micrographs. Representative TEM, SEM, and confocal micrographs were processed using FIJI (ImageJ) [26] using the original TIFF images.

Analysis of confocal microscopy micrographs was performed using scripts developed in Columbus analysis software (PerkinElmer, Waltham, MA, USA). Cell nuclei were segmented based on the DAPI signal and the cytoplasm segmented based on the Cell Mask Deep Red or cytoplasmic background DAPI signal. All border objects/cells were excluded from subsequent analyses. For measuring changes in GLP1R membrane localization, the cell membrane was segmented based on defining a percentage area relative to the boundary of the segmented cytoplasm. Mean fluorescence intensity for the GLP1R staining was measured from this cell membrane ROI. Fluorescence intensities were normalized to those measured from untreated cells and expressed as mean per cell per well. For colocalization analyses, Pearson’s correlation co-efficient was calculated for the correlation between the red and green channels and expressed as mean per cell per well. For all analyses, at least 16 images from triplicate experiments were included for each condition tested.

### 2.11. Data Analysis and Statistics

Graphs were prepared and curve fitting was performed using GraphPad Prism 8.

## 3. Results

### 3.1. Detection of Isotopically Labeled ASOs Delivered by Ligand-Mediated Endocytosis

We previously described the development of an ASO with 15× ^34^S incorporated in the PS backbone (^34^S_15_-ASO) [23]. A recent NanoSIMS study detected the cellular uptake of ^34^S-labelled ASOs; however, it did not attempt to measure ^34^S-ASO enrichment following ligand-mediated delivery [22]. To understand the applicability of ^34^S-ASO, we benchmarked our measurement in the method in hepatic spheroids with GalNAc-conjugated Malat1 ASO (GalNAc-ASO), which efficiently accumulated in hepatocytes [27]. We estimated the GalNAc-ASO endo-lysosomal concentration to be ~100 µM based on the number of ASGPRs present on a hepatocyte [28] and the approximate volume of the hepatocyte endosomal space [29]. The plot in Figure S1b shows that we measured a linear response through this concentration range of 2.6–260 µM using a standard material that mimicked the excess of ^34^S expected to be measured in the ^34^S-ASOcontaining organelles. Based on these data and the endogenous levels of ^34^S, we estimated our limit of detection for the ^34^S_15_-ASO to be ~20 µM. This was a significant improvement on the ~1mM LOD that we reported for the ^13^C labeling of drug molecules [21].

**Figure 1:**
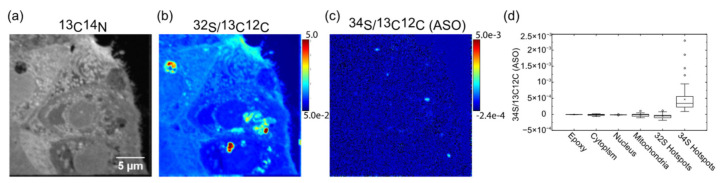
Detection of GalNAc-ASO in human hepatic spheroids; (**a**) ^13^C^14^N^-^ image of the perimeter of a hepatic spheroid incubated with GalNAc-^34^S-ASO (711nM) for 24H; (**b**) ^32^S^-^/^13^C^12^C^-^ ratio image of the spheroid; (**c**) ^34^S^-^/^13^C^12^C^-^ ratio image of the spheroid where the ^34^S^-^ was calculated as the excess respect to the average ^34^S^-^/^32^S^-^ across the total image and was scaled to the ASO by being divided by the number of ^34^S labels per ASO termed ^34^S^-^/^13^C^12^C^-^ (ASO). (**d**) ROI analysis expressed in ^34^S^-^/^13^C^12^C^-^ (ASO) for *n* = 7 treated hepatocytes analyzed.

Figure 1 shows a section of a primary human hepatic spheroid treated with 711nM GalNAc-^34^S_15_-ASO for 24 h. The ^13^C^14^N^-^ image (Figure 1a) provides the general structure of the cell in which large structures, such as nuclei and mitochondria, can easily be identified. The ^32^S/^12^C^13^C ratio image (Figure 1b) shows that the density of sulfur could vary by almost two orders of magnitude across the cell. This was problematic because the isotopic enrichment due to the ^34^S_15_-ASO was influenced by the local sulfur density. This meant that a small amount of ^34^S_15_-ASO would show high levels of enrichment in a region of low ^32^S density and, conversely, low enrichment in dense regions. To mitigate for this variation, we calculated the ^34^S counts in excess of the counts expected from the counts measured for ^32^S, and the average ratio measured across the whole image. This was very close to the reference Canyon Diablo Troilite CDT (^34^S/^32^S = 1/22.22 or 0.0450045). The excess ^34^S was then divided by the number of labels per ASO (15) and normalized to the carbon background (^13^C^12^C). We defined this scaled ratio as ^34^S/^13^C^12^C (ASO) (Equation (1)).

(1) S 34/C 13C 12 (ASO)=c 34ounts34−counts32×ratioave NLabels ×counts13C12C  

Figure 1c presents the image using the scaled signal (^34^S/^13^C^12^C (ASO)). The image shows that the ASO was detectable following the ligand-mediated uptake, where it was assumed to be located in the endosomal space. The box plots in Figure 1d show the value of ^34^S/^13^C^12^C (ASO) for several structures of interest as well as regions rich in ^32^S and ^34^S, showing that the receptor-mediated uptake was measurable using this labeling strategy.

### 3.2. Dual Labelling to Track Both Targeting Ligand and PS-ASO

**Figure 2:**
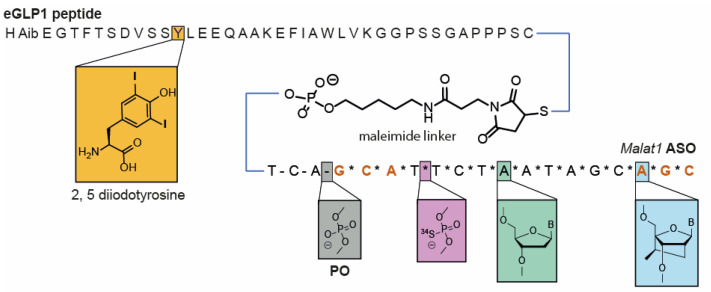
Schematic of double-labelled GLP1-ASO conjugate with maleimide linker.

To be able to measure both a targeting ligand and the ASO, we synthesized an eGLP1-ASO containing different labels on the *Malat1* ASO (15× ^34^S) and on eGLP1 (2× ^127^I) to generate ^127^I_2_-eGLP1-^34^S_15_-ASO (Figure 2). We validated this labeling strategy using a pseudo standard that we designed to approximate the atomic ratio of the ^127^I and ^34^S labels measured from three concentrations of ^127^I_2_-eGLP1-^34^S_15_-ASO conjugate (Figure S1a). We detected each label within the standard over a linear range from 2.6 to 260 µM (Figure S1b,c). We also determined that the signals measured from the equimolar standard for both labels had a linear relationship (Figure S1d).

**Figure 3:**
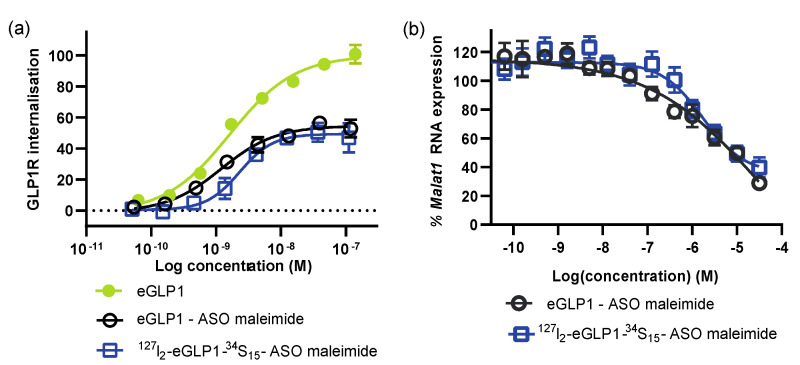
Verification of ^127^I_2_-eGLP1-^34^S_15_-ASO function. (**a**) GLP1R internalization assessed in U2OS cells using the PathHunter assay. (**b**) *Malat1* RNA expression in GLP1R-HEK293 cells after incubation with unlabeled or labeled eGLP1-ASO. Data shown as mean ± SEM for 3 independent experiments performed in duplicate.

We sought to verify that this dual labeling did not impede the eGLP1-ASO function. Both ^127^I_2_-eGLP1-^34^S_15_-ASO and the unlabeled eGLP1-ASO had similar activity in the PathHunter^®^ GLP1R-Activated GPCR Internalization U2OS Cell Line (Figure 3a). We observed reduced maximal activity but a similar potency for activating GLP1R internalization for both conjugates compared to eGLP1 (Figure 3a), in line with our previous work [10]. These eGLP1-ASO conjugates also demonstrated a comparable efficacy for reducing *Malat1* expression in GLP1R-HEK293 cells (Figure 3b). Overall, the incorporation of ^127^I_2_ and ^34^S_15_ labels did not appear to impede the eGLP1-ASO activation of GLP1R or Malat1 RNA knockdown, and this conjugate was suitable for studying eGLP1-ASO subcellular trafficking.

**Figure 4:**
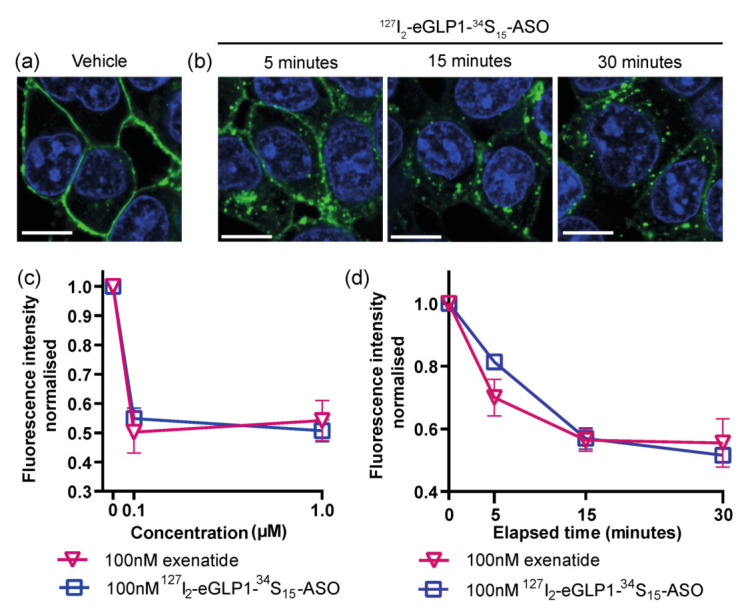
eGLP1-ASO-mediated GLP1R internalization in GLP1R-HEK293 cells. Representative confocal images of GLP1R localization in GLP1R-HEK293 cells following incubation with vehicle control (**a**) or ^127^I_2_-eGLP1-^34^S_15_-ASO (100 nM) for the time points indicated (**b**). Nuclei were counterstained with DAPI. (**c**) GLP1R mean fluorescence intensity was calculated from a region of interest encompassing the cell border. (**d**) GLP1R-HEK293 cells were incubated with ^127^I_2_-eGLP1-^34^S_15_-ASO or exenatide for 5–30 min at indicated concentrations and image analysis performed as in (**b**). For each condition, cells from at least 10 fields of view were analyzed in duplicate wells and data were expressed as mean ± SEM. Scale bar = 10 µm.

To understand ^127^I_2_-eGLP1-^34^S_15_-ASO internalization kinetics in GLP1R-HEK293 cells, we monitored eGLP1-ASO-induced GLP1R trafficking, since GLP1R has previously been shown to internalize together with agonists [11,12]. We employed the anti-GLP1R monoclonal antibody Mab7F38 [30], to detect GLP1R internalization by confocal microscopy (Figure 4a). Exenatide, a synthetic GLP1 analogue, served as a positive control for activating GLP1R internalization [13,14,15,31]. Under basal conditions, GLP1R was observed predominantly in the plasma membrane (Figure 4a). Following 30 min incubation with ^127^I_2_-eGLP1-^34^S_15_-ASO or exenatide, the GLP1R membrane fluorescence was reduced by ~50% (Figure 4b right panel and Figure 4c). GLP1R internalization was similar following exposure at 100 nM or 1 µM, indicating that the internalization saturated following exposure to 100 nM ^127^I_2_-eGLP1-^34^S_15_-ASO (Figure 4c). Next, we assessed the GLP1R internalization in GLP1R-HEK293 cells over time. We found that the GLP1R membrane fluorescence loss (Figure 4c) was initiated within 5 min and then plateaued within 15–30 min exposure to ^127^I_2_-eGLP1-^34^S_15_-ASO or exenatide (Figure 4b,d).

### 3.3. Identifying a Subcellular Compartment for eGLP1-ASO Localization

**Figure 5:**
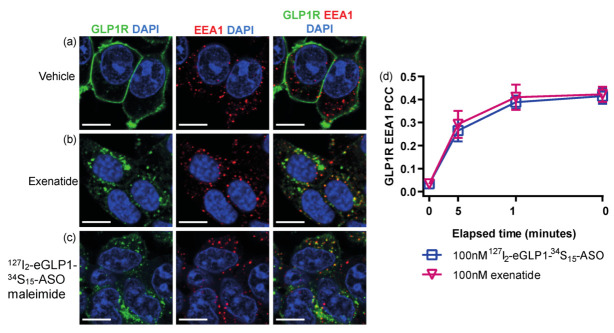
Representative confocal images of GLP1R and EEA1 following 30 min incubation with vehicle control (**a**), 100 nM exenatide (**b**), or 100 nM ^127^I_2_-eGLP1-^34^S_15_-ASO (**c**). Nuclei were counterstained with DAPI. Pearson’s correlation co-efficient for GLP1R colocalization with EEA1 was calculated from cytoplasmic ROIs (**d**). All analyses are presented as mean ± SEM for 3 independent experiments. For each condition, cells from at least 10 fields of view were analyzed from at least 2 technical replicates per experiment. Scale bar = 10 µm.

It has previously been shown that GLP1R agonists and GLP1R internalized together into early endosomes [11,12,15,31]. We performed confocal microscopy to assess GLP1R association with early endosomal-associated antigen 1 (EEA1), a marker of the early endosome. Under basal conditions, very little overlap was observed (Figure 5a), but GLP1R colocalization with EEA1 was observed, following incubation with exenatide (Figure 5b,d) or ^127^I_2_-eGLP1-^34^S_15_-ASO (Figure 5c,d).

**Figure 6:**
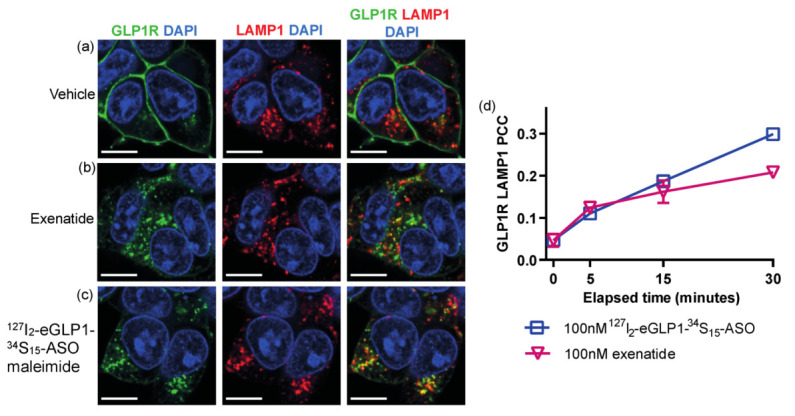
Representative confocal images of GLP1R and LAMP1 following 30 min incubation with vehicle control (**a**), 100 nM exenatide (**b**), or 100 nM ^127^I_2_-eGLP1-^34^S_15_-ASO (**c**). Nuclei were counterstained with DAPI. Nuclei were counterstained with DAPI. Pearson’s correlation co-efficient for GLP1R colocalization with LAMP1 was calculated from cytoplasmic ROIs (**d**). All analyses are presented as mean ± SEM for 3 independent experiments. For each condition, cells from at least 10 fields of view were analyzed from at least 2 technical replicates per experiment. Scale bar = 10 µm.

GLP1R localization with the late endosome marker LAMP1 (lysosome-associated membrane protein 1) was also examined and under basal conditions, and very little overlap between GLP1R and LAMP1 was observed (Figure 6a). There was an increase in the GLP1R colocalization with LAMP1 after 30 min exposure to ^127^I_2_-eGLP1-^34^S_15_-ASO (Figure 6c,d) or exenatide (Figure 6b,d). Overall, our data indicated that GLP1R and, therefore, ^127^I_2_-eGLP1-^34^S_15_-ASO should be located in endosomes after 30 min exposure.

**Figure 7:**
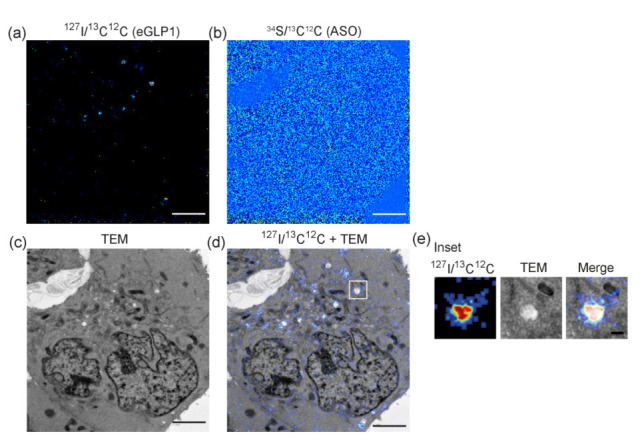
(**a**) ^127^I/^13^C^12^C image of an HEK293 cell treated with 1 µM ^127^I_2_-eGLP1-^34^S_15_-ASO for 30 min. (**b**) *^34^*S/^13^C^12^C (ASO) image of the same cell. (**c**) TEM micrograph of the same cell. (**d**) TEM/^127^I/^13^C^12^C image overlay. (**e**) Zoom of inset in (**d**) to show a single endosome containing ^127^I_2_-eGLP1. Scale bar = 3 µm. Scale bar inset = 300 nm.

We next sought to detect ^127^I_2_-eGLP1 and ^34^S_15_-ASO following a 30 min exposure of GLP1R-HEK293 cells to ^127^I_2_-eGLP1-^34^S_15_-ASO, and correlate the NanoSIMS signal to TEM micrographs. Again, we presented the ASO image with the unit *^34^*S/^13^C^12^C (ASO), while the eGLP1 image was generated by scaling the ^127^I counts by the number of labels per eGLP1 and normalizing it to the carbon image (^13^C^12^C^-^) to generate ^127^I/^13^C^12^C (eGLP1). The NanoSIMS image showed puncta for ^127^I-eGLP1 (Figure 7a) that were not observed in the ^34^S-ASO image (Figure 7b). Overlaying the ^127^I-eGLP1 image onto the TEM micrograph from the same section (Figure 7c) revealed that the ^127^I-eGLP1 signal colocalized with transparent structures, presumably endosomes (Figure 7d,e, Figures S2 and S3).

### 3.4. Quantification of Both Targeting Ligand and Drug Moiety

**Figure 8:**
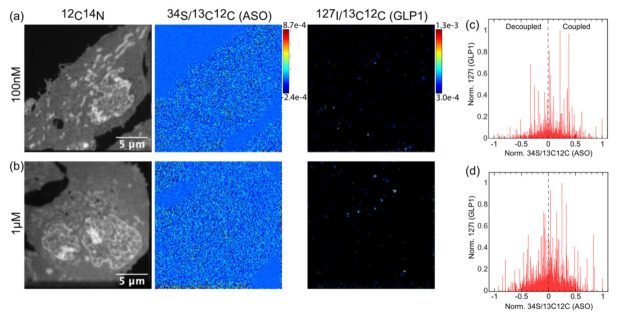
NanoSIMS images of GLP1R-HEK293 cells incubated for 30 min with 100 nM or 1 µM ^127^I_2_-eGLP1-^34^S_15_-ASO maleimide. (**a**) SIMS images of 100 nM incubation GLP1R-HEK293 cells,^12^C^14^N, *^34^*S/^13^C^12^C (ASO), ^127^I/^13^C^12^C(GLP1). (**b**) SIMS images of 1 mM incubation GLP1R-HEK293 cells,^12^C^14^N, *^34^*S/^13^C^12^C (ASO), ^127^I/^13^C^12^C(GLP1). (**c**) ROI analysis 100 nM incubation GLP1R-HEK293 cells. (**d**) ROI analysis 1mM incubation GLP1R-HEK293 cells.

The images in Figure 8 show GLP1R-HEK293 cells incubated with either 100 nM (a) or 1 mM (b) ^127^I_2_-eGLP1-^34^S_15_-ASO for 30 min. The *^34^*S/^13^C^12^C (ASO) images did not show clear punctate structures when compared to the ^127^I/^13^C^12^C (eGLP1) images at either incubation concentration. We noted that the incubation concentration also had very little effect on the intensity of the ^127^I_2_-eGLP1 measurement, suggesting that the uptake was saturated mirroring the saturation of GLP1R internalization observed in Figure 4b.

The evaluation of the relative amounts of ^127^I_2_-eGLP1 and ^34^S_15_-ASO was complicated by the nature of the ratio image, which was used to generate the *^34^*S/^13^C^12^C (ASO) image. Briefly, because there was a ratio measurement registered at almost all of the ~65000 pixels in the image, probability dictated that 10,400 pixels would have values above 1σ. Therefore, a small number of pixels with a true value of 1 or even 2σ above the average became lost in the distribution of the background, as observed in the ^34^S/^13^C^12^C (ASO) images. To address the complexity of the data, we binned each image down to 468 × 468 nm, which was slightly larger than the structures observed in the ^127^I/^13^C^12^C(GLP1) image, and then normalized to the maximum value. These data were plotted with respect to the normalized ^34^S/^13^C^12^C (ASO) for each ROI, for both the 100 nM (Figure 8c) and 1 µM (Figure 8d) incubations, respectively. If we assumed a strong coupling between ^127^I-eGLP1 and ^34^S-ASO, then we would expect the normalized ^127^I/^13^C^12^C(GLP1) values to be heavily shifted to the right. However, the ^127^I/^13^C^12^C(GLP1) values appeared to be fairly equally distributed, with a few higher values distributed towards the right (Figure 8d). Next, we sorted ^127^I/^13^C^12^C(eGLP1) ROIs containing (3σ cut off) into two classes. The first class comprised ^127^I/^13^C^12^C(eGLP1) ROIs with a positive ^34^S/^13^C^12^C (ASO) signal, which represented ROIs containing an intact eGLP1-ASO conjugate (coupled). The second class comprised ^127^I/^13^C^12^C(eGLP1) ROIs with a negative ^34^S/^13^C^12^C (ASO) signal, representing a decoupled eGLP1-ASO conjugate. We analyzed 54 coupled and 44 decoupled ROIs for the 100 nM treatment (*n* = 3 cells) and 144 coupled and 123 decoupled ROIs for the 1 mM treatment (*n* = 3 cells).

### 3.5. Increasing the Stability of the Linker between eGLP1 and ASO Increases Colocalization of Conjugate Components and Reduces RNA Knockdown Efficacy

**Figure 9:**
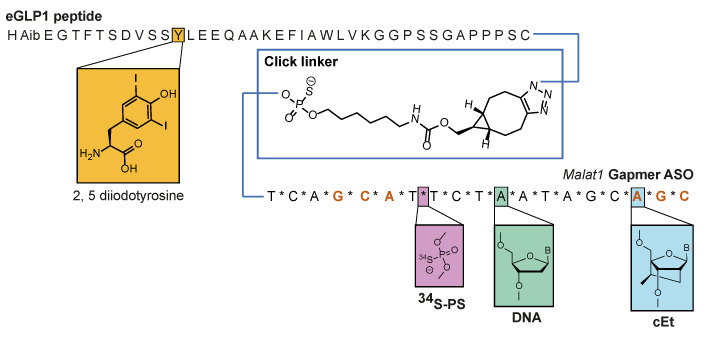
Schematic of double-labelled GLP1-ASO conjugate with click linker.

To further investigate this potential decoupling, we compared ^127^I_2_-eGLP1-^34^S_15_-ASO (which contained a maleimide linker) with a more chemically stable conjugate, ^127^I_2_-eGLP1-^34^S_19_-ASO click (Figure 9). The latter conjugate contained a hydrolysis-resistant click linker and the TCA portion of the sequence was equipped with more nuclease-stable PS linkages labelled with ^34^S. Therefore, the ^34^S incorporation increased from 15 to 19 providing 27% more signal per ASO. This increase in ^34^S labels was accounted for when calculating *^34^*S/^13^C^12^C (ASO). We also increased the section thickness from 300 nm to 1 μM to enable more extensive sampling, which caused the TEM imaging to be unfeasible.

**Figure 10:**
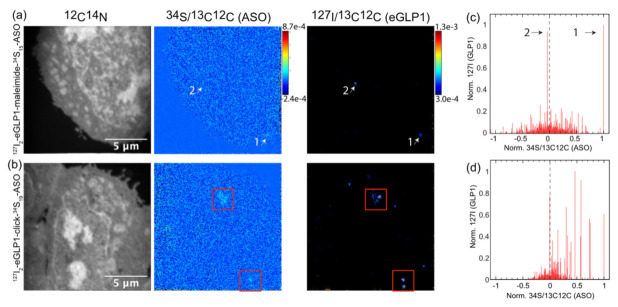
The ^127^I_2_-eGLP1-^34^S_19_-ASO click. (**a**) NanoSIMS images of GLP1R-HEK293 cells incubated for 30 min with ^127^I_2_-eGLP1-^34^S_15_-ASO maleimide: ^12^C^14^N, *^34^*S/^13^C^12^C (ASO), ^127^I/^13^C^12^C (eGLP1). (**b**) NanoSIMS images of GLP1R-HEK293 cells incubated with ^127^I_2_-eGLP1-^34^S_19_-ASO click: ^12^C^14^N, *^34^*S/^13^C^12^C (ASO), ^127^I/^13^C^12^C(GLP1). White arrows indicate spots 1 and 2 as described in the text. Red boxes indicate spots of ^34^S/^13^C^12^C (ASO), which were observed together with spots of ^127^I/^13^C^12^C (eGLP1). (**c**) ROI analysis of ^127^I_2_-eGLP1-^34^S_15_-ASO maleimide-treated GLP1R-HEK293 cells with signal corresponding to spots 1 and 2 indicated by black arrows. (**d**) ROI analysis of ^127^I_2_-eGLP1-^34^S_19_-ASO click-treated GLP1R-HEK293 cells.

The second analysis of ^127^I_2_-eGLP1-^34^S_15_-ASO maleimide uptake in GLP1R-HEK293 cells showed similar findings to the first, with 19 coupled and 16 decoupled ROIs observed (*n* = 3 cells). The measurement in Figure 10a shows two very distinct *^127^*I/^13^C^12^C (eGLP1) spots of similar intensity, marked as one and two in the NanoSIMS image, which were also visible in the plot in Figure 10c. The ^127^I/^13^C^12^C (eGLP1) spot one was correlated with the highest ^34^S/^13^C^12^C (ASO) value, and ^127^I/^13^C^12^C (eGLP1) spot two showed no enrichment of ^34^S/^13^C^12^C (ASO), indicating decoupling. This strongly suggested that that the sensitivity of the sulfur measurement was not the underlying cause of the apparent decoupling. The eGLP1 detected in spot one was in fact more strongly coupled to ASO than the eGLP1 detected in spot two.

Increasing the linker stability within the ^127^I_2_-eGLP1-^34^S_19_-ASO click conjugate clearly had an impact on the colocalization of ^34^S/^13^C^12^C (ASO) and ^127^I/^13^C^12^C (eGLP1). This was highlighted in Figure 10b by the red squares, in which ^34^S/^13^C^12^C (ASO) spots could be more clearly observed compared to the ^127^I_2_-eGLP1-^34^S_15_-ASO maleimide images in Figure 10a. However, the ROI analysis in Figure 10d showed a more nuanced picture. We did not observe the expected shift towards the right, indicating that the click-conjugated ASO was still coupled to eGLP1, but we could also observe very intense ^127^I/^13^C^12^C (eGLP1) ROIs lacking corresponding ^34^S/^13^C^12^C (ASO), indicating some separation of the ASO from eGLP1. Overall, for the ^127^I_2_-eGLP1-^34^S_19_-ASO click conjugate incubation, we analyzed 45 coupled and 19 decoupled ROIs (*n* = 3 cells). A more robust coupling was observed for the ^127^I_2_-eGLP1-^34^S_19_-ASO click compared to the ^127^I_2_-eGLP1-^34^S_15_-ASO maleimide (19 coupled and 16 decoupled).

Finally, we assessed the *Malat1* expression levels by qPCR following the incubation of GLP1R-HEK293 cells with ^127^I_2_-eGLP1-^34^S_19_-ASO click and observed a decreased *Malat1* knockdown compared to ^127^I_2_-eGLP1-^34^S_15_-ASO maleimide (Figure S4). This suggested that the preferential endosomal escape of ASO we measured for ^127^I_2_-eGLP1-^34^S_15_-ASO maleimide was a critical step in the productive delivery of this ASO.

## 4. Discussion

Here, we used NanoSIMS imaging to quantify both components of a ligand-PS-ASO conjugate in subcellular compartments of intact cells, paving the way for the better understanding of mechanisms underlying enhanced ASO activity following ligand-mediated delivery. We previously employed ^13^C labeling for the subcellular detection of a drug, but this approach suffered from a low limit of detection. In the current study, we instead used the ^34^S labeling method to detect PS-ASO [23] in combination with a novel approach to detect the eGLP1-targeting ligand, which was synthesized to incorporate diiodotyrosine, allowing for the detection of ^127^I-eGLP1 by NanoSIMS. We first evaluated the ^34^S-ASO uptake in hepatic spheroids incubated with GalNac-^34^S-ASO and observed accumulation in subcellular structures. Based on previous studies, we hypothesized that these were components of the endolysosomal pathway [32]. We developed quantification methodology for ^34^S-ASO and evaluated this in hepatic spheroids incubated with GalNac-^34^S-ASO, providing a proof of concept for the detection of ASO following ligand-mediated delivery to cells.

The double-labelled eGLP1-ASO that we synthesized maintained function, both in promoting GLP1R internalization and in knocking down the target *Malat1* RNA. Intriguingly, our NanoSIMS images of ^127^I-eGLP1 and ^34^S-ASO revealed that very few endosomes containing ^127^I-eGLP1 also contained enough ^34^S-ASO to provide a measurable enrichment of ^34^S. This suggested that the ^34^S-ASO delivered by eGLP1 preferentially escaped the endosome within 30 min of exposure to GLP1R-HEK293 cells. Most of the current knowledge about ASO subcellular trafficking and endosomal escape results from studies of unconjugated or ‘naked’ PS-ASO uptake in permissive cell lines, and may not translate to eGLP1-ASOs, given that PS-ASOs can be taken up by gymnosis, and not necessarily via classical clathrin-mediated endocytosis [18]. However, naked PS-ASO efficacy is not typically correlated with PS-ASO uptake, and is instead thought to be related to mechanisms of subcellular trafficking [33,34,35,36], supporting the idea that conjugation to eGLP1 drives the ASO into productive pathways.

The majority of studies point to the late endosome or early to late endosome transition as the critical location for ASO escape [36,37,38,39]. While it was not possible to distinguish early and late endosomes in our EM images, we did observe eGLP1-ASO conjugate-stimulated GLP1R colocalization with markers of both the early and late endosomes by confocal microscopy. Previous estimations of ASO endosomal escape were based on studies of short interfering RNA (siRNA), which reported 1.5–3.5% of siRNA escape from early endosomes [40,41]. Our data suggested that 1.5–3.5% endosomal escape was an underestimation for ASOs delivered by eGLP1-mediated endocytosis, and may explain why eGLP1-ASOs are so potent in vivo [10]. Perhaps our most exciting finding was that increasing the stability of the conjugate produced a measurable retention of the ^34^S-ASO, which corresponded to a reduction in knockdown (Figure S4). This result provided a potential mechanism underlying an observed reduction in potency for a GLP1 conjugate utilizing the click linker for knocking down *Malat1* in vivo [17]. The preferential escape of the ASO could be an advantage of targeting strategies that exploit GPCRs for cellular delivery. Furthermore, we showed that the stability of the connection between the ASO and GPCR ligand is a tunable parameter, which can be used to affect the ASO productive uptake. The ability to evaluate linker stability presents an interesting opportunity to track the lifetime of the conjugate through a time-dependent pulse chase experiment, or to employ correlative imaging to evaluate the state of the conjugate in specific endosomal compartments. Identifying where and when the ASO exits within the endosomal space would provide mechanistic insights into the efficacy of ASO therapeutics.

## Data Availability

Data are available upon request.

## Supplementary Materials

The following are available online at https://www.mdpi.com/article/10.3390/pharmaceutics14020463/s1, Figure S1: validation of labeling strategy, Figure S2: GLP1 signal localization to endosome-like structures in HE293 cells following 30 min incubation of 100 nM ^127^I_2_-glp1-maleimide-^34^S_15_MALAT1, Figure S3: GLP1 signal localization to endosome-like structures in HEK293 cells following 30 min incubation 1 µM ^127^I_2_-glp1-maleimide-^34^S_15_MALAT1, Figure S4: (A) *Malat1* knockdown following incubation with either eGLP1-click-*Malat1* ASO or eGLP1-maleimide-*Malat1* ASO as measured by qPCR.
