# Supplementary material for: NanoSIMS Imaging Reveals the Impact of Ligand-ASO Conjugate Stability on ASO Subcellular Distribution
Source: Pharmaceutics. 2022 Feb 21;14(2):463. doi: 10.3390/pharmaceutics14020463 (PMC8876276; doi:10.3390/pharmaceutics14020463)
Supplement: Supplementary file 1 [file pharmaceutics-14-00463-s001.zip › pharmaceutics-1533827-supplementary.pdf]

pharmaceutics

Supplementary Materials: NanoSIMS Imaging Reveals the Impact of Ligand-ASO Conjugate Stability on ASO Subcellular Distribution

Rouven Stulz, Cécile Becquart, Jelena Lovric, Carolina Tängemo, Aurélien Thomen, Dzenita Bazdarevic, Neda Najafinobar, Anders Dahlén, Anna Pielach, Julia Fernandez-Rodriguez, Roger Strömberg, Carina Ämmälä, Shalini Andersson and Michael E. Kurczy

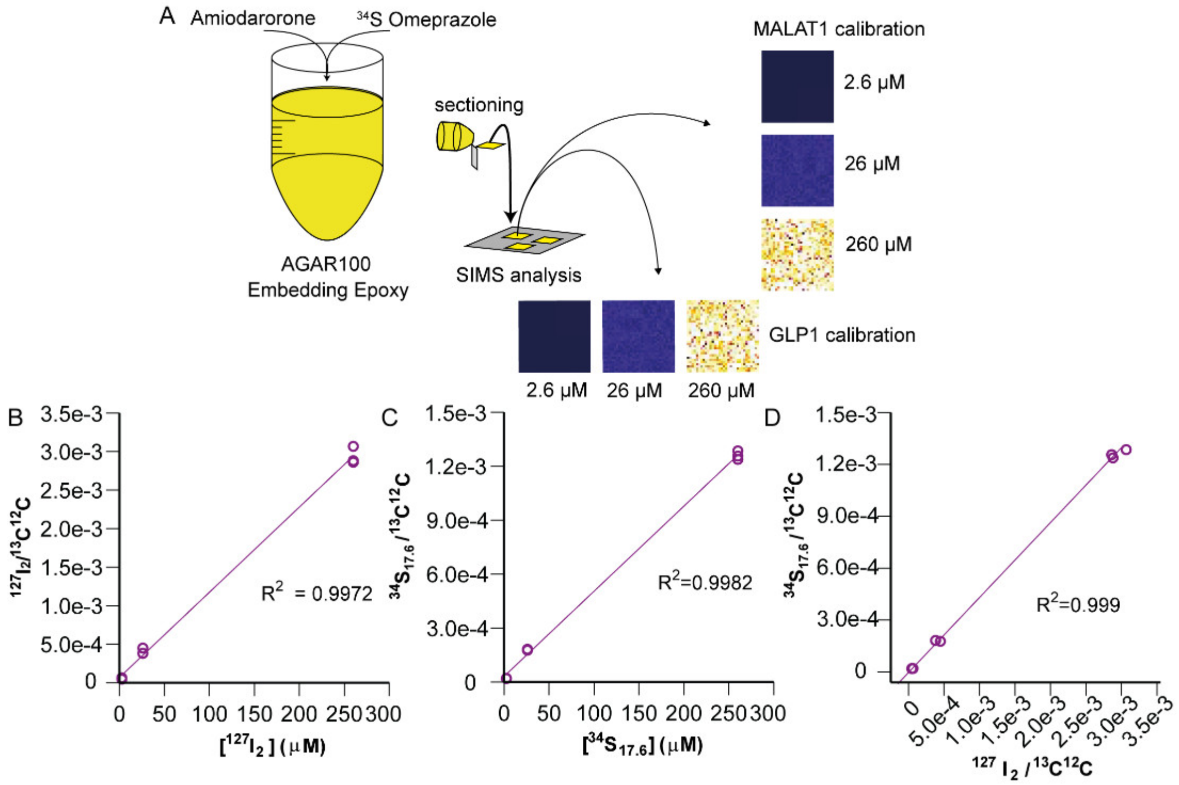

Figure S1. Validation of labeling strategy. (**A**) Schematic of the preparation of the pseudo double labelled standard and calibration curve generation using iodine containing amiodarone and ^{34}S labelled Omeprazole. (**B**) ^{127}I (pseudo eGLP1) calibration curve. (**C**) ^{34}S (pseudo ASO) calibration curve. (**D**) Signal correlation for equimolar pseudo dual labelled standard.

The calibration curve was generated from an epoxy standard with an ^{34}S:^{127}I ratio of 8.8:1. A 30 ul Dimethylacetamide solution containing 8.7 mM Amiodarone (2 moles ^{127}I) and 153 mM ^{34}S labelled Omeprazole (1 mole ^{34}S) as added to 1 ml of AGAR100 and cured 3 days at 50º. After curing, the sample was sectioned and mounted on 5 X 5 mm Si wafers. This is equivalent to an ASO conjugate with the following labelling strategy (17.6 ^{34}S ASO to 2 ^{127}I GLP1).

The precision of an individual ratio measurement is estimated using a Poisson relationship by finding the inverse of the square root of the numerator for the $^{34}S/^{32}S$ (counts of ^{34}S) for an ROI. For example a 16X16 ROI rich in sulphur will typically have 45000 counts of $^{13}C^{12}C$, 22500 counts ^{32}S and 1012 counts of ^{34}S. The numerator for the sulphur ratio give a precision of approximately 3%. **The limit of detection** is defined as the lowest

measurable enrichment distinguishable from the control. In the case of an image the control is the surrounding non enriched regions. Therefore the Poisson uncertainty also serves as a limit of detection, meaning that to detect the labelled ASO the isotopic ratio needs to be increased by 3%. By increasing the expected counts of ^{34}S by 3% (1042 in the example above) and plugging all the count measurements into equation 1 we find the ^{34}S ASO the uncertainty and limit of detection is 4.4e-5 $^{34}S/^{13}C^{12}C$ (ASO). Likewise, a less sulphur rich ROI of the same size with 45000 counts of $^{13}C^{12}C$ 2250 counts ^{32}S and 101 counts of ^{34}S, would have an uncertainty of 10% but interestingly because of the endogenous sulphur is low the uncertainty and LOD is reduced to 1.5e-5. $^{34}S/^{13}C^{12}C$ (ASO). In practice the precision and the LOD is better than 1e-4 $^{34}S/^{13}C^{12}C$ (ASO) for a typical ROI, which corresponds to 17 μM using the calibration curve in figure S1.

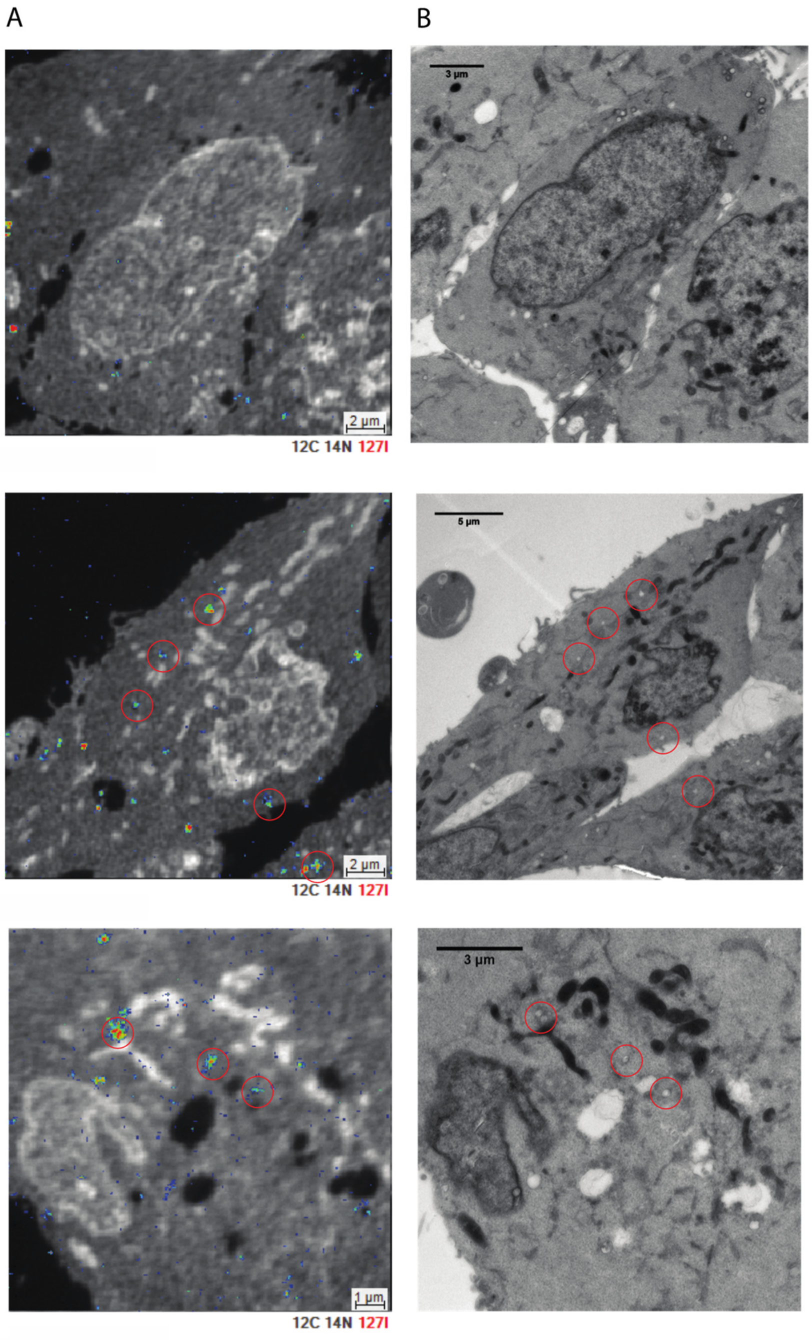

Figure S2. GLP1 signal localization to endosome like structures in HEK293 cells following 30 min incubation of 100 nM $^{127}I_2$-glp1-maleimide-$^{34}S_{15}$MALAT1. Column A: Sims image overlay of ^{127}I and $^{12}C^{14}N$ showing the subcellular distribution of Iodine labeled GLP1. Column B. Corresponding TEM images, red circles indicate transparent subcellular structures (taken to be endosomes) which correlate to iodine rich structures identified by SIMS imaging.

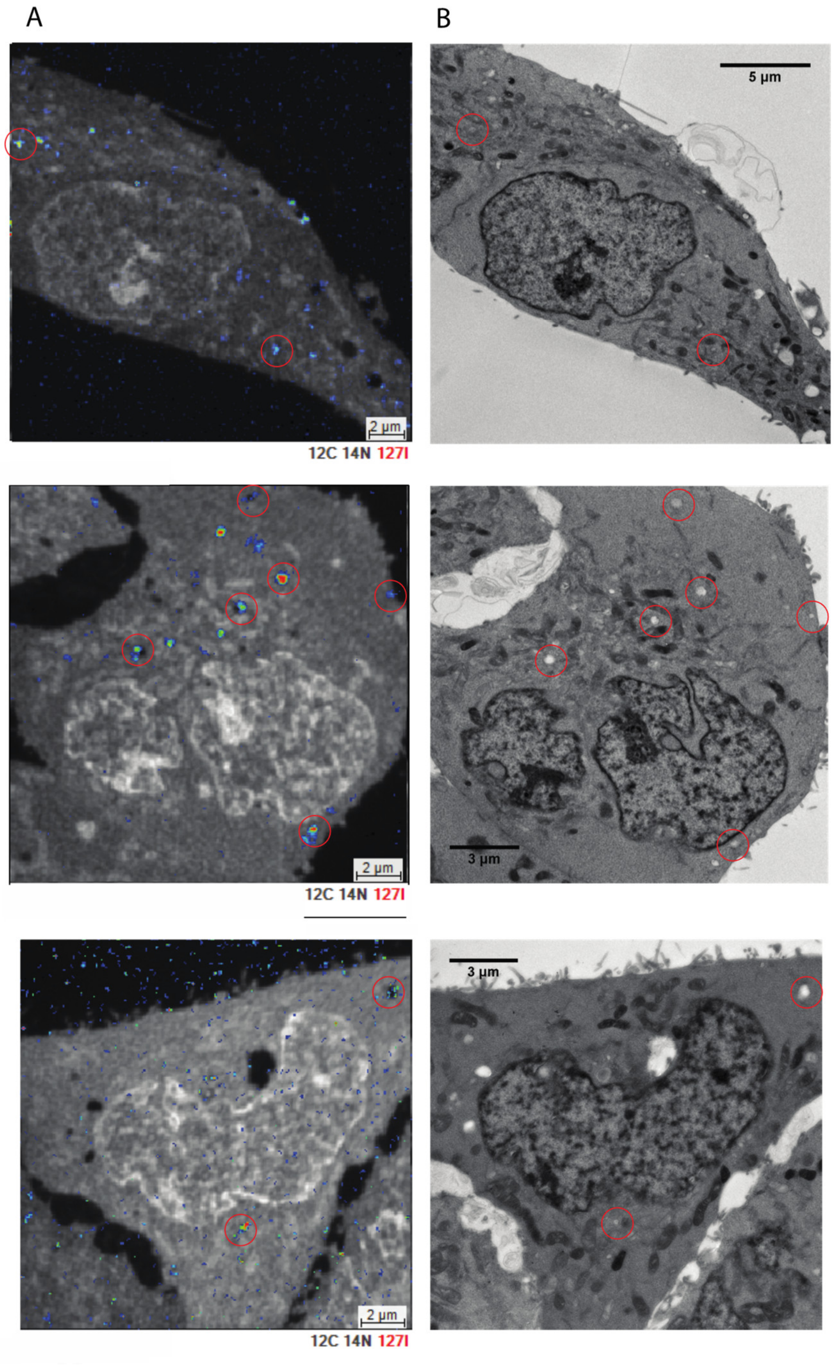

Figure S3. GLP1 signal localization to endosome like structures in HEK293 cells following 30 min incubation 1 µM $^{127}I_2$-glp1-maleimide-$^{34}S_{15}$MALAT1. Column A: Sims image overlay of ^{127}I and $^{12}C^{14}N$ showing the subcellular distribution of Iodine labeled GLP1. Column B. Corresponding TEM images, red circles indicate transparent subcellular structures (taken to be endosomes) which correlate to iodine rich structures identified by SIMS imaging.

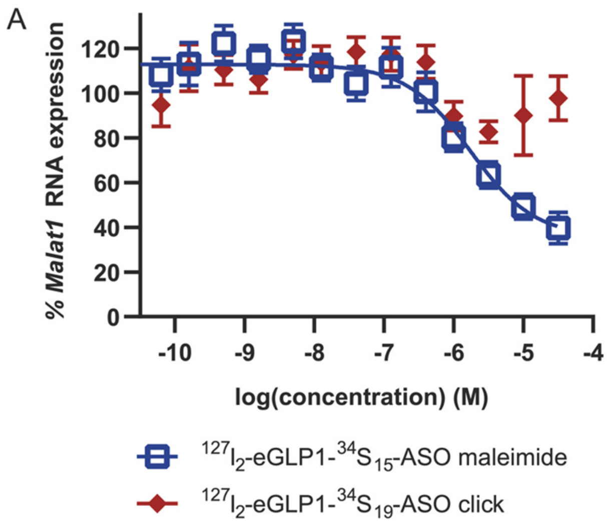

Figure S4. (A) *Malat1* knockdown following incubation with either eGLP1-click-*Malat1* ASO or eGLP1-maleimide-*Malat1* ASO as measured by qPCR. Data are presented as the mean ± SEM of 3 independent experiments.
